# Habitat patch size alters the importance of dispersal for species diversity in an experimental freshwater community

**DOI:** 10.1002/ece3.2858

**Published:** 2017-06-17

**Authors:** Matthew S. Schuler, Jonathan M. Chase, Tiffany M. Knight

**Affiliations:** ^1^ Department of Biology Washington University in St. Louis St. Louis MO USA; ^2^ German Centre for Integrative Biodiversity Research (iDiv) Halle‐Jena‐Leipzig Germany; ^3^ Institute for Computer Science Martin Luther University Halle‐Wittenberg Halle Germany; ^4^ Institute of Biology Martin Luther University Halle‐Wittenberg Halle Germany; ^5^ Department of Community Ecology Helmholtz Centre for Environmental Research‐UFZ Halle Germany; ^6^ Present address: Darrin Fresh Water Institute Department of Biology Rensselaer Polytechnic Institute Troy NY 12180

**Keywords:** diversity, ENS_PIE_, fragmentation, habitat size, patch connectivity

## Abstract

Increased dispersal of individuals among discrete habitat patches should increase the average number of species present in each local habitat patch. However, experimental studies have found variable effects of dispersal on local species richness. Priority effects, predators, and habitat heterogeneity have been proposed as mechanisms that limit the effect of dispersal on species richness. However, the size of a habitat patch could affect how dispersal regulates the number of species able to persist. We investigated whether habitat size interacted with dispersal rate to affect the number of species present in local habitats. We hypothesized that increased dispersal rates would positively affect local species richness more in small habitats than in large habitats, because rare species would be protected from demographic extinction. To test the interaction between dispersal rate and habitat size, we factorially manipulated the size of experimental ponds and dispersal rates, using a model community of freshwater zooplankton. We found that high‐dispersal rates enhanced local species richness in small experimental ponds, but had no effect in large experimental ponds. Our results suggest that there is a trade‐off between patch connectivity (a mediator of dispersal rates) and patch size, providing context for understanding the variability observed in dispersal effects among natural communities, as well as for developing conservation and management plans in an increasingly fragmented world.

## 1 Introduction

Over the past several decades, community ecology has shifted from a focus on local factors such as abiotic filters and species interactions, to one that recognizes the interplay between local and regional factors, such as dispersal (e.g., Leibold et al., 2004; Logue, Mouquet, Peter, & Hillebrand, 2011). For example, isolated habitat patches are expected to have fewer species than well‐connected habitat patches, because isolation reduces the dispersal rates of species among habitat patches (Andrén, 1994; Hanski, 2005; MacArthur & Wilson, 1963; Prugh, Hodges, Sinclair, & Brashares, 2008; Strantford & Robinson, 2005). In addition to understanding variation in richness among habitats that naturally vary in their isolation (e.g., serpentine soils; Harrison, 1999; small ponds; Chase, 2003), interest in the influence of habitat isolation on dispersal limitation has been boosted by global habitat loss and fragmentation, leading to species extinctions and reduced biodiversity (Damschen et al., 2008; Gilbert, Gonzalez, & Evans‐Freke, 1998; Gonzalez & Chaneton, 2002; Haddad et al., 2015; Helm, Hanski, & Pärtel, 2006).

Classical ecological theories predict that dispersal often positively affects local species richness (Leibold et al., 2004; MacArthur & Wilson, 1963; May, Giladi, Ziv, & Jeltsch, 2012), and meta‐analyses of experimental results have generally supported those predictions (Cadotte, 2006; Logue et al., 2011; Myers & Harms, 2009). However, the magnitude and direction of the effects of dispersal on local species richness vary greatly among studies. Variation even exists in the same experimental system. For example, in small, freshwater ponds, some studies have shown that increased dispersal rates have a negligible influence on local species richness (e.g., Forbes & Chase, 2002; Shurin, 2000) while others have shown strong effects (Cottenie & De Meester, 2004; Howeth & Leibold, 2010; McCauley, 2006). Some studies have even shown that increased dispersal or connectivity can have negative effects on species richness or diversity (reviewed in: Debinski & Holt, 2000; Mouquet & Loreau, 2003; Cadotte, 2006; Myers & Harms, 2009; Åström & Pärt, 2013).

Several mechanisms have been proposed explaining the disparity of results among studies assessing the role of dispersal for patterns of species richness. These mechanisms include differences in species' traits and life histories (Öckinger, Franzén, Rundlöf, & Smith, 2009; Prugh et al., 2008; Thomas, 2000), priority effects (Shurin, 2000), abiotic constraints such as drought and habitat disturbance (Hoyle & Gilbert, 2004; Östman, Kneitel, & Chase, 2006), predators (Kneitel & Miller, 2003; Shurin, 2001), environmental heterogeneity (Cottenie & De Meester, 2004; Matthiessen, Mielke, & Sommer, 2010), and habitat size (Krauss, Klein, Steffan‐Dewenter, & Tscharntke, 2004; Öckinger et al., 2010; Steffan‐Dewenter, 2003). Research on the effects of habitat isolation and habitat patch size is important for understanding how local species richness (i.e., within patch) will be affected as habitats across the globe become smaller and more isolated. One problem with many of these studies, however, is that habitat area and heterogeneity are often highly correlated (e.g., Öckinger et al., 2010). Therefore, assessing the simultaneous effects of patch size and dispersal on local species richness patterns is difficult (Hanski, 2005), because differences in heterogeneity could affect species richness patterns. Of course, some studies have attempted to understand these processes in natural systems (e.g., Haddad et al., 2015; Simberloff & Wilson, 1970; Warren, 1996), but controlled experiments that test the effects of habitat size and dispersal are rare (e.g., Gonzalez & Chaneton, 2002; Rantalainen, Fritze, Haimi, Pennanen, & Setälä, 2005).

Large habitat patches typically have more species than small habitat patches as a result of both regional and local processes (Hanski, 1999; Lomolino, 2000; MacArthur & Wilson, 1963; Simberloff, 1976). For example, large habitat patches typically have increased habitat heterogeneity, which would support more types of species, and lower rates of extinction due to larger population sizes of rare species (Connor & McCoy, 1979; Cornell, 2013; MacArthur & Wilson, 1963; Rosenzweig, 1995; Simberloff & Wilson, 1970). One theory regarding dispersal predicts that large areas are more likely to contain a greater number of species than smaller areas simply due to random chance (i.e., the target effect; Turner, 1989). Alternatively, species may not disperse randomly or may be dispersal limited (e.g., Jacquemyn, Butaye, & Hermy, 2001). In such cases, landscape factors including increased habitat heterogeneity, habitat patch proximity, or trophic interactions would affect the relationship between patch size and local species richness within a metacommunity (Scheffer et al., 2006).

In small habitat patches, species more strongly compete for limited resources (Tilman, 1994), and face a greater risk of extinction due to small population sizes and edge effects (Bender, Contreras, & Fahrig, 1998; Collingham & Huntley, 2000; Hill, Hastings, & Botsford, 2002; Quinn & Hastings, 1987)). Therefore, we might predict that increased dispersal from the regional species pool should allow species in small habitat patches to persist that otherwise might not be able to (e.g., rescue effects) (Brown & Kodric‐Brown, 1977; Eriksson, Elías‐Wolff, Mehlig, & Manica, 2014; Gonzalez, Lawton, Gilbert, Blackburn, & Evans‐Freke, 1998; MacArthur & Wilson, 1963; Thompson, Rayfield, & Gonzalez, 2017). Alternatively, increased dispersal might impede competition–colonization trade‐offs that limit the presence of competitively dominant species in small habitats. The increased presence of competitive species in small habitats would lead to a reduction in species richness in small habitats (Mouquet & Loreau, 2003; Tilman, 1994).

Increased dispersal could also negatively effect the amount of species turnover among habitat patches in a metacommunity (i.e., species homogenization; Gilbert et al., 1998; Loreau, 2000; Kneitel & Miller, 2003). Reduced species turnover among habitat patches occurs because the size of the regional species pool is limited and most species do not occupy all patches in a metacommunity. Therefore, increased dispersal could increase the percent of patches occupied by each species (Loreau, 2000), reducing differences in composition among habitat patches (e.g., Cottenie, Michels, Nuytten, & De Meester, 2003; Forbes & Chase, 2002). If highly competitive species are dispersal limited, then increasing the occupancy of those species among patches would lead to a reduction in local species richness (Mouquet & Loreau, 2002). Given this scenario, as the patch occupancy of species increases, poor competitors surviving in small habitat patches would likely go extinct. Therefore, increased dispersal might reduce species richness in small habitat patches, and increase species richness in large habitat patches.

To investigate how habitat size and dispersal rates interactively affect patterns of species richness, we manipulated a diverse community of zooplankton (crustaceans and rotifers) in mesocosms that mimic freshwater ponds. Due to the relationship between habitat size and extinction rate, we expected larger mesocosms to have lower extinction rates than smaller mesocosms (MacArthur & Wilson, 1963). Large mesocosms may also have more available niche space for new species to become established (Cornell, 2013; Rosenzweig, 1995). Therefore, dispersal would increase species richness in large mesocosms, and dispersal would have a null or negative effect in small mesocosms due to increased competition and higher extinction rates. This would especially be true if dominantly competitive species obtain an advantage due to increased dispersal, and further increase the extinction rate in small mesocosms. Alternatively, if the available niche space does not depend on habitat size, and competitive species are not dispersal limited, then we would expect to find that higher rates of dispersal would increase species richness more in small mesocosms than in large. In this case, we expect that rescue effects would be an important mechanism affecting the number of species in small mesocosms. Indeed, our results indicate that high rates of dispersal increased species richness more in small mesocosms, but had negligible effects in larger mesocosms. These results are consistent with predictions from the Equilibrium Theory of Island Biogeography model.

## 2 Methods

### 2.1 Experimental design

In May of 2013, we arranged small (300 L) and large (900 L) mesocosms in an old field at Washington University's Tyson Research Center (Eureka, Missouri, USA). For this experiment, we had five replicates of each of the four treatments (two levels of habitat size and two levels of dispersal) (twenty total mesocosms). Four of the five replicates were placed in a fully dispersed array, with each treatment represented equally in each row and column across the mesocosm array. The fifth replicate of each treatment was placed in another row, separated from the main array because there was an old structure in the field. Each treatment was present in this row, in case there was a bias from the structure. The arrangement of the mesocosms was meant to distribute treatments across any systematic biases that could have been present in the field (e.g., shading, temperature, etc.).

Large and small habitats naturally differ in their inherent geometric properties. To reduce the confounding effects of these geometric properties (e.g., surface area) had on our study, we used small mesocosms that were oblong (oval), and large mesocosms that were round. Without the shape adjustment, the surface area of the water in the small mesocosms would have been proportionally greater (per volume) than the large mesocosms. The estimated surface area of the small mesocosms was 6670 cm^2^, and the surface area of the large mesocosms was 22,070 cm^2^. Therefore, the ratio of surface area between large and small was 3:10, very similar to the 1:3 volume ratio that was used for the experiment. Therefore, the amount of sunlight (i.e., energy) entering the large and small mesocosms was approximately proportional, although large mesocosms would receive more total energy than small mesocosms. Using small, oval mesocosms also allowed for us to use similar depths in the large and small mesocosms. The depth in the small mesocosms was 57 cm, and the depth in the large mesocosms was 54 cm. Schuler, Chase, and Knight (2015) used the same mesocosms, with equal depths used in this study to investigate how energy input (manipulated by shade cloth density) and habitat size interacted to affect zooplankton communities.

On 20 May, we filled each mesocosm in a systematic order (by number, not by treatment) with water from a nearby well. We ran the well for 3 hr to clear any unwanted sediments. Additionally, to ensure that variation in well conditions did not influence the water quality in the mesocosms, all mesocosms were filled 50%, before we continued to fill each mesocosm to the desired level. Filling tanks half full, in order of arrangement and not by treatment, ensured that each treatment received an equal amount of variation in initial water quality. Four days after filling the mesocosms, inorganic nitrogen (in the form of NaNO_3_) and phosphorus (in the form of Na_3_PO_4_) were introduced so that initial total dissolved nitrogen (TN) was ~1,600 μg/L and total dissolved phosphorus (TP) was ~100 μg/L (16N:1P). These values would represent a eutrophic state, if all of the phosphorus were immediately biologically available, and mesocosms were open systems (see Carlson, 1977). However, these are closed systems with few phosphorus inputs, and not all of the phosphorus is biologically available. Due to high iron content of the well water, some of the phosphorus binds to iron to form ferrous phosphate (Fe_3_O_8_P_2_), which would not readily release useable phosphorus under normal conditions in these mesocosms (Baldwin and Williams 2007). The nutrient addition was repeated 45 days after zooplankton were inoculated, to ensure that sufficient nutrient levels were maintained to sustain algal growth for the duration of the experiment (Hall, Smith, Lytle, & Leibold, 2005).

To create a model regional species pool for our experiment, we collected zooplankton from eight ponds near Tyson Research Center, which were known to have high zooplankton diversity and variable composition. A dense zooplankton stock was obtained by filtering water from the eight ponds using an 80‐μm zooplankton net. To establish sufficient populations of zooplankton in each mesocosm prior to dispersal treatments, on 10 June we inoculated each mesocosm with 200 ml of the water with concentrated densities of zooplankton (consisting of approximately 4,000 individuals) into each small mesocosm and 600 ml of the water (approximately 12,000 individuals) into each large mesocosm. Stocking these densities ensured that enough individuals of each species were present in each mesocosm. In a previous study, we found that these stocking densities maintained a diverse community of zooplankton in mesocosms for at least 90 days (Schuler et al., 2015). We stocked three times as many zooplankton in the large mesocosms compared to small mesocosms, because the large mesocosms had three times the amount of water. Therefore, the initial densities of zooplankton in the large and small mesocosms were equal. We removed invertebrate predators from the zooplankton slurry using dissecting trays, to avoid biases caused by introducing different numbers of predators into large or small mesocosms. Additionally, to stop oviposition from insects and frogs, as well as minimize the dispersal of zooplankton beyond the manipulations of the experiment, each mesocosm was covered with a polyurethane mesh, with a mesh size of 0.85 × 0.85 mm (Howeth & Leibold, 2010).

Models of dispersal and habitat size assume that individuals disperse randomly (e.g., MacArthur & Wilson, 1963). To test how different rates of random dispersal affected species richness patterns in differently sized habitats, we filtered zooplankton from the same eight ponds that the initial stock of zooplankton were taken from. We dispersed individuals into the mesocosms twice; 7 and 21 days after the initial zooplankton were stocked in the mesocosms (17 June and 1 July, respectively). In the high‐dispersal treatment, we dispersed the same proportion of individuals originally stocked in the mesocosms (~4,000 in small mesocosms; ~12,000 in large mesocosms). For the low‐dispersal treatment, the zooplankton stock was diluted so that 10 ×  fewer individuals were dispersed (~400 in small mesocosms, and ~1,200 in large mesocosms). These dispersal events represent natural forms of dispersal for zooplankton in metacommunities of ponds, where water flow among ponds disperses individuals at rates dependent upon the distance to the nearest pond, and the intensity of precipitation events (e.g., Michels, Cottenie, Neys, & De Meester, 2001).

### 2.2 Data collection

On 1 September, ninety days after the initial zooplankton stock was added to the mesocosms, we sampled the zooplankton community to understand how dispersal and habitat size interacted to affect species richness. During the experiment, zooplankton species may have aggregated in the mesocosms. To ensure that we effectively sampled the species pool present in each mesocosm and treatment, we collected and filtered water from a variety of locations and depths in each mesocosm. Using string, we divided small mesocosms into two equally sized sections, and each large mesocosm into six equally sized sections (keeping the number of samples proportional to mesocosm size). We then collected a total of 18 L from each section of every mesocosms. To obtain each 18 L sample, we used a 3‐L pitcher, tipped it upside down, and dunked it into the tank to the desired depth. We then turned the pitcher right side up, collecting the zooplankton from that location without heavily disturbing the rest of the mesocosm. We repeated this collection technique six times per section in each mesocosm, moving to a different location or depth each time. For example, if one sample was collected near the surface of the water, we collected the next sample from 20 to 45 cm. We collected six 18 L samples from each large mesocosm, and two 18 L samples from each small mesocosm. We filtered three times more water from the large mesocosms compared to the small mesocosms to maintain proportionality with mesocosm size. To condense each sample into a 50‐ml centrifuge tube for future enumeration, we used an 80‐μm zooplankton net, and immediately preserved each sample with Lugol's iodine for later identification and enumeration under a Nikon 400‐mm dissecting scope (Dodson, Arnott, & Cottingham, 2000). In total, forty‐nine species of zooplankton were identified, including thirty‐four rotifers, eight cladocerans, six copepods, and one ostracod.

### 2.3 Analyses

To identify and enumerate zooplankton, we gently mixed each 50 ml sample and extracted 10 ml, to count the number of individuals and the number of species (mean = 379 individuals, *SE* = 23.8). We saved the remaining portion of the sample for future analyses. We used these methods based on a previous study, where we verified that 20% of each sample sufficiently represented the number of species and density of species in each mesocosm (Schuler et al., 2015). These data allowed us to estimate the abundance of individuals in each sample (individuals 18 L^−1^), and the density of species per sample (species richness 18 L^−1^). Sampling species per unit area can reduce the probability of detecting rare species in large areas compared to small areas (the habitat per se effect, Connor & McCoy, 1979). To account for under‐sampling of rare species, extrapolated species richness was estimated using Chao's (1984) nonparametric method for extrapolating the total number of species in a sample. Chao's (1984) estimator allows for the comparison of species richness values given the possibility that sampling efficacy differs among treatments, by estimating the number of potential missed species that results from under‐sampling (Chao, Colwell, Lin, & Gotelli, 2009; Colwell et al., 2012; Gimaret‐Carpentier, Pélissier, Pascal, & Houllier, 1998). If the estimated number of species from each mesocosm does not differ significantly from the measured number of species, or differs equally among treatments, then there were no sampling biases from each treatment.

Species richness as a comparative metric of treatment effect is highly biased by sample size, and the size of the species pool (Chase & Knight, 2013; Lande, 1996). Therefore, to compare species richness values among treatments, we used Hurlbert's Probability of Interspecific Encounter (PIE), which gives an estimate of the evenness of the relative abundance distribution, and therefore acts as a sample size independent method of rarefaction (Hurlbert, 1971). Hurlbert's PIE indicates differences in the relative abundances of species among treatments, by defining the slope of the rarefaction curve at its base (Lande, 1996; Olszewski 2004, Dauby & Hardy, 2012; Chase & Knight, 2013). PIE is equatable to Simpson's index (where Simpson's index is D, and PIE is 1‐D). To diversity, we converted PIE to an “Effective Number of Species” (ENS_PIE_; $1/\sum_{i=1}^{S}p_{i^{2}}$ ), where *S* represents the number of species, and *p*_i_ is the proportion of the community comprised of species *i* (Jost, 2006). Using ENS_PIE_ allows one to compare the relative abundance distributions among treatments (Chase & Knight, 2013; Dauby & Hardy, 2012). Additionally, ENS_PIE_ allows one to disentangle sampling effects (e.g., the More Individuals Effect; Srivastava & Lawton, 1998), from treatment effects that would alter the coexistence mechanisms of species in the experiment. If species richness and ENS_PIE_ change unidirectionally among treatments, then we can infer that coexistence mechanisms differ among those treatments. Alternatively, a change in species richness without a corresponding change in ENS_PIE_ could indicate that the differences in species richness resulted from changes in the total abundances of species, but not their relative abundances (i.e., a sampling effect).

For each sample, extrapolated species richness (Chao, 1984) was calculated using the *estimateR* function, and ENS_PIE_ values were calculated using *diversity* function in the Vegan Package in R (Oksanen et al., 2011). ANOVA with dispersal and size as independent variables was used to compare the response of the observed species richness, extrapolated species richness, species' abundance, and diversity (ENS_PIE_), with Tukey's Honestly Significant Difference (HSD) post hoc tests for multiple comparisons.

The primary focus of this study was to investigate how increased species dispersal affected the number of species in communities, and how habitat size mediated the relationship between species dispersal and species richness. However, we also examined whether dispersal or habitat size influenced species composition among treatments. To do this, we compared Bray–Curtis dissimilarities among large and small mesocosms, as well as low‐ and high‐dispersal mesocosms using permutation‐based ANOVA (PERMANOVA). We use nonmetric multidimensional scaling to graphically display compositional differences in zooplankton species composition among the treatments (e.g., Knapp, Matthews, & Sarnelle, 2001). To understand which species could be responsible for any differences found in composition among the treatments, we used a Similarity Percentages (SIMPER) analysis. This allowed us to determine whether the species that affected compositional differences had particular traits that would have been affected by the rate of dispersal. We also assessed which species changed in their relative abundances the most among the high‐ and low‐dispersal treatments, which would offer insight in to the mechanisms that led to differences in species richness or diversity. If the relative abundance of mostly rare species increased, then we could infer that “rescue effects” played a role in affecting species richness and diversity differences among the treatments. If common species increased or decreased in their abundances, then dispersal may have altered composition–colonization trade‐offs, which would affect the coexistence mechanisms of species in the mesocosms. We calculated Bray–Curtis dissimilarity matrices using the *veg.dist* function in R, and PERMANOVA was conducted using the *adonis* function in R (Oksanen et al., 2011). For the Similarity Percentages Analysis, we used the *simper* function in the Vegan Package in R (Oksanen et al., 2011).

## 3 Results

**Figure 1:**
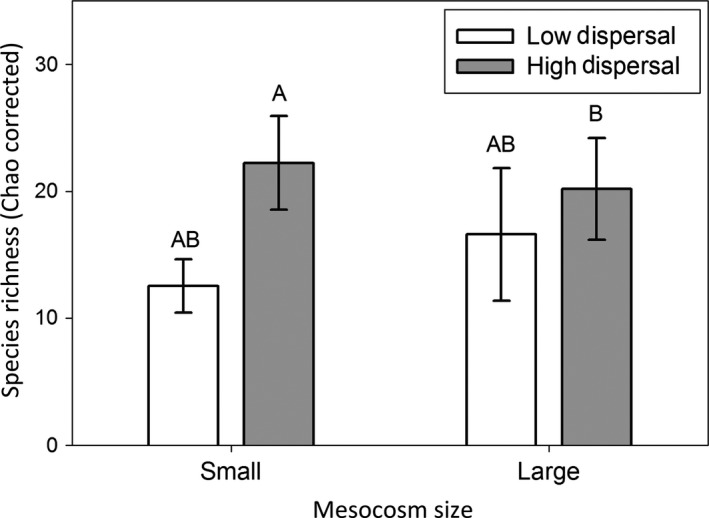
Species richness per sample (18L of water; Chao corrected), in large and small mesocosms with high and low rates of dispersal. Letters indicate significant differences among treatments.

**Figure 2:**
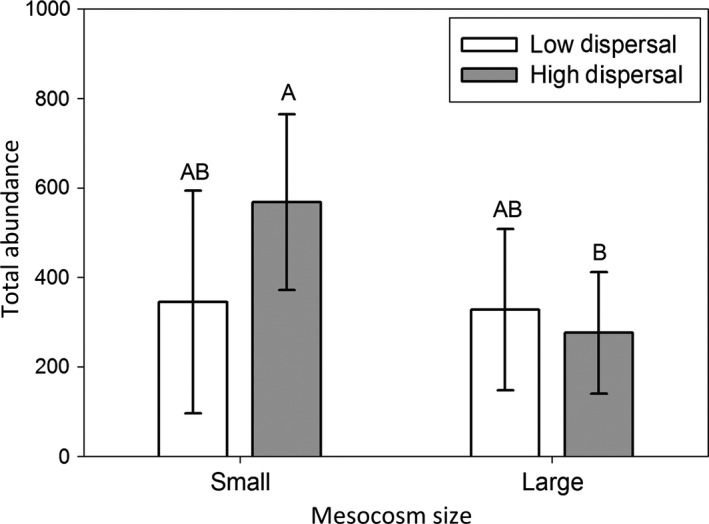
The abundance of individuals per sample (18L of water), in large and small mesocosms with high and low rates of dispersal. Letters indicate significant differences among treatments.

**Figure 3:**
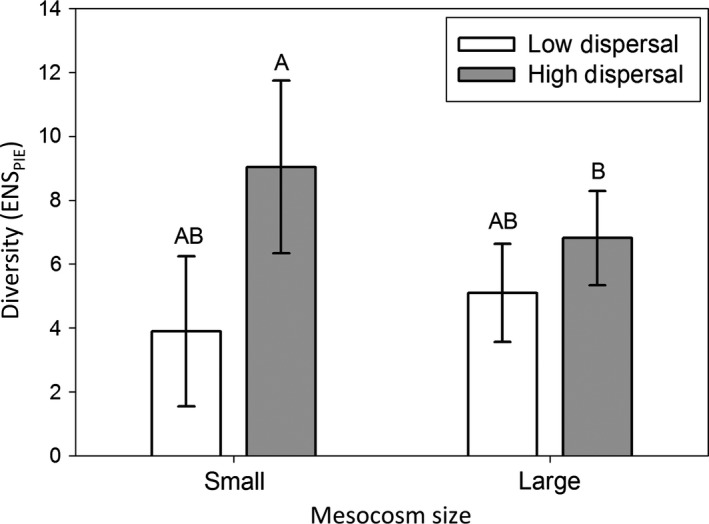
Species diversity per sample (ENS_PIE_; 18L of water), in large and small mesocosms with differing rates of dispersal. Changes in ENS_PIE_ indicate a shift in the relative abundances of species, as ENS_PIE_ is a metric of evenness. Letters indicate significant differences among treatments.

Species richness (Chao corrected number of species 18 L^−1^) was affected by dispersal, as well as the interaction between habitat size and dispersal, but species richness was not affected by habitat size (Table 1a, Figure 1); the positive effect of dispersal on species richness was significant in small mesocosms (*p* < .001) but was not significant in large mesocosms (*p* = .313). The total abundance was affected by habitat size but not dispersal, and the interaction was significant (Table 1b, Figure 2). However, Tukey's HSD post hoc test revealed that total abundance was not significantly different among any of the pairwise treatments (*p* > .10). The effective number of species measured from PIE (ENS_PIE_) per sample mirrored the species richness results, where dispersal increased ENS_PIE_ (i.e., increased community evenness) in small habitats (*p* < .001), but not large habitats (*p* = .398) (Table 1c; Figure 3).

**Table 1:** ANOVA tables for the effects of habitat size and dispersal rate

Treatment	*df*		*F* value	*p* value
(a) Extrapolated species richness (18 L^−1^) (Chao)				
Size	1		0.518	0.482
Dispersal	1		22.09	<0.001
Size × dispersal	1		7.034	0.045
Residuals	16			
(b) Species' abundances (18 L^−1^)				
Size	1		4.855	0.042
Dispersal	1		1.497	0.238
Size × dispersal	1		3.858	0.067
Residuals	16			
(c) Species diversity (ENS_PIE_; 18 L^−1^)				
Size	1		0.46	0.507
Dispersal	1		20.888	<0.001
Size × dispersal	1		5.228	0.036
Residuals	16			

ANOVA tables a–c correspond to Figures 1, 2, 3, respectively.

**Figure 4:**
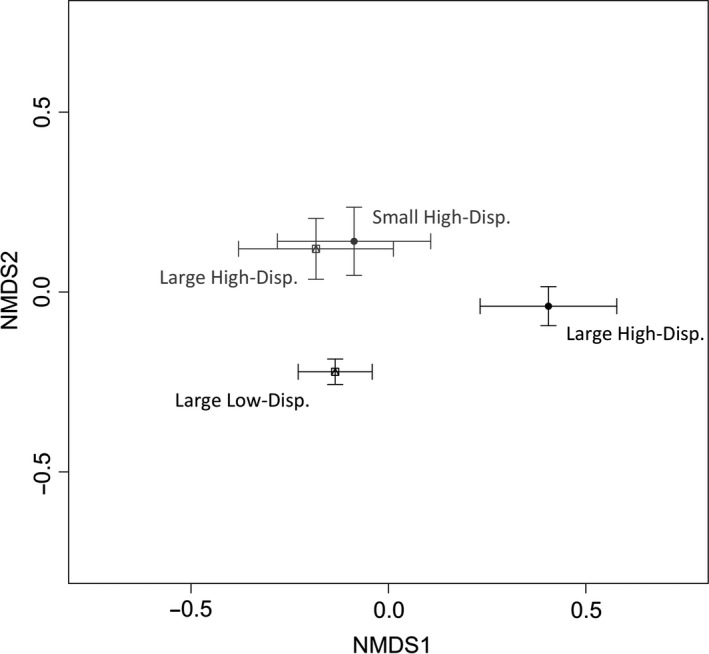
Nonmetric Multidimensional Scaling plot showing the Bray–Curtis dissimilarities among treatments. Gray plots indicate high‐dispersal communities, black plots indicate low‐dispersal communities, filled circles represent small mesocosms, and squares represent large mesocosms. The center of each plot represents the centroid of the cluster of Bray–Curtis dissimilarity values for each treatment, and the error bars represent one standard deviation from the centroid. Stress = 0.21

PERMANOVA on the Bray–Curtis dissimilarities showed that the main and interactive effects of habitat size and dispersal significantly contributed to compositional differences among communities (Table 2, Figure 4). Large and small, high‐dispersal mesocosms were compositionally more similar than the large and small low‐dispersal mesocosms (Figure 4). The SIMPER analysis showed that common and rare species, but mostly rotifers contributed to the compositional shifts in large and small mesocosms, as well as in the high‐ and low‐dispersal treatments (Tables S1, S2). Two of the rare species (*Monostyla closterocerca* and *Monostyla bulla*) and two of the common species (*Philodina* spp. and *Platyias patulus*) showed the greatest percent shifts in their relative abundances in the small high‐dispersal, compared to small low‐dispersal treatments (Table S3).

**Table 2:** Results from PERMANOVA, using Bray–Curtis dissimilarities to test for compositional differences among dispersal and size treatments

Treatment	*df*	χ^2^	*F* value	*p* value
Size	1	0.272	2.560	.01
Dispersal	1	0.323	3.038	.01
Size × dispersal	1	0.196	1.844	.04
Residuals	16	1.701		

## 4 Discussion

Our results indicate that although dispersal rates play an important role in determining species richness and composition patterns, these effects are contingent upon the context in which the dispersal takes place. Specifically, we found a strong positive effect of dispersal in smaller habitat patches, and no effect of dispersal in larger habitats patches (Figures 1, 3, Table 1). Although these patterns are consistent with theoretical expectations (e.g., MacArthur & Wilson, 1963; May et al., 2012), few empirical studies have been able to examine the interaction of habitat size and dispersal rate in affecting species richness, due to confounding factors like trophic interactions, or the relationship between habitat size and heterogeneity (but see Simberloff & Wilson, 1970; Warren, 1996; Myers & Harms, 2009). In this study, we found that higher dispersal rates led to higher species richness and that this increase was due to dispersal increasing the evenness (ENS_PIE_) of a community rather than due to a “More Individuals Effect” (Srivastava & Lawton, 1998). Likely, the increase in evenness resulted because dispersal buffered rare species from extinction, although changes in competition may have played an important role as well.

As predicted by metacommunity theory (Gonzalez et al., 1998; May et al., 2012), the composition of species between small and large mesocosms with high dispersal was more similar than the composition of species between small and large mesocosms with low dispersal (Figure 4, Table 2). These compositional shifts were expected, because when a regional species pool remains constant, and the average number of species supported in each habitat patch of a metacommunity increases, those communities will become more similar to one another (Howeth & Leibold, 2010). We determined that the homogenization effect occurred in both small and large mesocosms, likely due to species replacement in large mesocosms, and rescue effects in small mesocosms. The fact that species richness and diversity did not change in large, high‐dispersal mesocosms, but the composition of species shifted to be more similar to the small, high‐dispersal mesocosms, suggests that the addition of species from the regional species pool led to the replacement of some species in large, high‐dispersal mesocosms. Some species replacement may have also occurred in small mesocosms. However, new species also established in the small, high‐dispersal mesocosms, as indicated by an increase in ENS_PIE_ (a metric of evenness) and an increase in species richness. We found evidence that common species decreased their abundances in the small, high‐dispersal mesocosms, and rare species increased in abundance (Table S3). Changes in composition–colonization trade‐offs could have affected the common species, and rescue effects could have benefited the rare species. These two effects are likely inter‐related. Abundant species are likely more competitive for limiting resources, and a reduction in their abundance due to newly established species competing for resources may allow rare species an opportunity to increase in abundance, especially with additional propagules.

The results of this study provide one way to understand variation in the degree to which communities are dispersal limited. Of course, there are also several alternative and inter‐related mechanisms that likely influence how dispersal affects species richness. For example, the size of the regional species pool, the total abundance of individuals in local habitats (i.e., mass effects), or the characteristics of the species that are being dispersed could all alter the way dispersal affects patterns of diversity (Altermatt, Schreiber, & Holyoak, 2011; Gravel, Mouquet, Loreau, & Guichard, 2010). In some circumstances, increased dispersal rates could decrease the likelihood of coexistence and diversity. For example, if a highly competitively species benefits from dispersal, and moves into previously unoccupied habitats, the increased competition could reduce species richness by competitive exclusion (Calcagno, Mouquet, Jarne, & David, 2006; Gravel et al., 2010; Levine, 2000; Mouquet & Loreau, 2003; Tilman, 1994). The mesocosms in this experiment were established in early summer, and some zooplankton species could have been missing from the regional species pool at that time, because the composition of zooplankton species often shifts during summer months. Thus, it is possible that future dispersal events could act to reduce diversity if dispersal allowed competitively dominant species to enter the mesocosm (Matthews, Cottee‐Jones, & Whittaker, 2014). However, such negative effects of dispersal should be most apparent in small habitats, where the total populations of rare species are small. Because we found increased species richness and diversity in the small mesocosms, relative to the large mesocosms, we surmise that the competitively dominant species did not benefit from dispersal in this experiment.

The scope of this project was to examine the role that habitat size plays in mediating the effects of increased dispersal on species richness and diversity, but can say little about the specific mechanisms by which these outcomes occurred. Although we attempted to minimize the confounding abiotic and biotic differences caused by inherent geometric differences between large and small mesocosms, some differences might have affected the results of our study. For example, differences between small and large mesocosms likely altered the effect of abiotic constraints (e.g., temperature) on the zooplankton community, which in turn could alter the species traits favored by these conditions, as well as the nature of interspecific interactions among the species, all of which would require more detailed study. The experimental mesocosms used in this study were relatively homogeneous in their environmental conditions, but small temperature differences exist between large and small mesocosms (<2°C). We do not have evidence that differences in temperature among small and large mesocosms altered the competition or coexistence of zooplankton. The average daily high temperature in small and large mesocosms (~21 and ~23°C, respectively) is consistent with temperatures experienced by coexisting zooplankton in natural ponds and lakes (e.g., Gilbert & Hampton, 2001).

Understanding the specific demographics of species that differ in their relative abundances could give insight into how generalist species compared to specialist species respond to the addition of individuals in size‐limited habitats. In this study, rare species benefited from dispersal in small habitats and common species seemed to be negatively affected (Table S3). These data suggest that increased dispersal resulted in a “rescue effect,” where rare species benefited from dispersal and were therefore able to overcome Allee effects and/or stochastic extinction events that would have otherwise reduced species richness or diversity in small mesocosms (Amarasekare, 1998; Fowler, 2009; Leibold et al., 2004). Additionally, some common species were negatively affected, potentially from increased competition of species that were dispersed into the mesocosms. Most of the species that contributed to differences in composition, and experienced large changes in their relative abundances were rotifers. The only cladoceran that contributed to compositional differences was *Chydorus sphaericus*, which is a relatively small cladoceran and prone to competition effects from larger cladocerans like *Simocephalus vetulus* and *Daphnia* spp. (Dodson, 1974). If large cladocerans and copepods benefited from increased dispersal, their increased presence in small mesocosms may have negatively affected some rotifers, especially *Philodina* spp. which is a small rotifer lacking any defensive spines. This is because large cladocerans and some copepod species directly consume small rotifer species, which can limit their abundances (Williamson & Butler, 1986). Some of the rare species that increased in abundance were rotifer species like *Monostyla bulla* and *Monostyla closterocerca*. These species may be better adapted to dealing with mostly planktonic predatory copepods, as they spend a majority of their time near the edge of the mesocosms (Nagata & Hanazato, 2006). However, species like *Philodina* spp. and *Platyias patulus* are more planktonic, and would be exposed to increased predation risks (Nagata & Hanazato, 2006).

We intentionally limited the number of trophic interactions in this study to examine how habitat size alters the effects of dispersal on species richness and diversity in the absence of potentially confounding effects from predators (e.g., Chase, Burgett, & Biro, 2010; Shurin, 2000). However, in more natural systems, the effect of predators on species richness is likely to be influenced by the size of the habitat patch (Petermann et al., 2015). As a result, we might expect an even more complex interaction between habitat size and dispersal on community‐level patterns when a more intact food web is present.

Our results add an interesting perspective to growing body of research assessing the effects of dispersal on species richness and diversity (reviewed by Cadotte, 2006; Myers & Harms, 2009; Grainger & Gilbert, 2016). As habitat patches become smaller and more isolated, increased dispersal rates among the remaining fragments in a metacommunity should often increase the number of species supported by each patch. However, our results suggest that the positive effects of dispersal on species richness and diversity will be much stronger in smaller, relative to larger habitat patches. This makes sense in the light of metacommunity theory (e.g., MacArthur & Wilson, 1963; May et al., 2012) and suggests that there is a trade‐off between habitat size and connectivity with regard to patterns of diversity and composition; the influence of dispersal decreases as habitat size increases. Our research indicates that understanding the variation in the responses of communities to variation in dispersal rates is important, especially as habitats become more fragmented and isolated.

## Conflict of interest

None declared.

## Author Contributions

MSS, TMK, and JMC conceived and designed the experiment. MSS performed the experiment, and collected and analyzed the data. MSS, TMK, and JMC wrote the manuscript. MSS and TMK provided funding support.
