# Supplementary material for: Habitat patch size alters the importance of dispersal for species diversity in an experimental freshwater community
Source: Ecol Evol. 2017 Jun 17;7(15):5774–83. doi: 10.1002/ece3.2858 (PMC5551274; doi:10.1002/ece3.2858)
Supplement: Supplementary file 1 [file ECE3-7-5774-s001.docx]

**Supplementary Material:**

Table S1. SIMPER analysis examining the percent contribution (and its standard deviation, SD) of different species to the main effect of dispersal treatment (high vs. low dispersal) on species composition in mesocosms.

| Species Name | % Contribution | SD % Contribution |
| --- | --- | --- |
| *Philodina spp.* | 10.25 | 11.06 |
| *Platyias patulus* | 9.82 | 8.63 |
| *Chydorus sphaericus* | 5.27 | 4.9 |
| *Monostyla bulla* | 4.27 | 5.95 |

Table S2. SIMPER analysis examining the percent contribution (and its standard deviation, SD) of different species to the main effect of habitat size on species composition in large and small mesocosms.

| Species Name | % Contribution | SD % Contribution |
| --- | --- | --- |
| *Philodina spp.* | 12.31 | 12.96 |
| *Platyias patulus* | 8.83 | 8.34 |
| *Chydorus sphaericus* | 5.71 | 5.61 |
| *Monostyla bulla* | 3.63 | 5.65 |

Table S3. The effect of dispersal rate (low vs. high) on the four species that showed the biggest relative shifts in abundance in the small mesocosms. Two common species in the low dispersal treatment were more rare in the high dispersal treatment whereas two rare species in the low dispersal treatment were more common in the high dispersal treatment.

| Species Name | Low (Rel. Abund.) | High (Rel. Abund.) |
| --- | --- | --- |
| *Philodina spp.* | 0.2566 | 0.008 |
| *Platyias patulus* | 0.199 | 0.066 |
| *Monostyla closterocerca* | 0.026 | 0.072 |
| *Monostyla bulla* | 0.0092 | 0.082 |
